# Advance care planning in Parkinson’s disease: ethical challenges and future directions

**DOI:** 10.1038/s41531-019-0098-0

**Published:** 2019-11-22

**Authors:** Leonard L. Sokol, Michael J. Young, Jack Paparian, Benzi M. Kluger, Hillary D. Lum, Jessica Besbris, Neha M. Kramer, Anthony E. Lang, Alberto J. Espay, Ornella M. Dubaz, Janis M. Miyasaki, Daniel D. Matlock, Tanya Simuni, Moran Cerf

**Affiliations:** 1 The Ken and Ruth Davee Department of Neurology, Northwestern University Feinberg School of Medicine, Chicago, IL USA; 2 McGaw Bioethics Scholars Program, Center for Bioethics and Humanities, Northwestern University Feinberg School of Medicine, Chicago, IL USA; 3 Department of Medicine, Northwestern University Feinberg School of Medicine, Chicago, IL USA; 4 Department of Neurology, Massachusetts General Hospital, Harvard Medical School, Boston, MA USA; 5 Independent Researcher, San Francisco, CA USA; 6 Division of Neuropallaitive Care, Department of Neurology, University of Colorado School of Medicine, Aurora, CO USA; 7 Eastern Colorado VA Geriatric Research Education and Clinical Center and the Division of Geriatric Medicine, Department of Medicine, University of Colorado School of Medicine, Aurora, CO USA; 8 Departments of Neurology and Supportive Care Medicine, Cedars-Sinai Medical Center, Los Angeles, CA USA; 9 Departments of Medicine (Section of Palliative Medicine) and Neurology, Rush University School of Medicine, Chicago, IL USA; 10 The Edmond J. Safra Program in Parkinson’s Disease and the Morton and Gloria Shulman Movement Disorders Clinic, University Health Network, University of Toronto, Toronto, ON Canada; 11 Gardner Family Center for Parkinson’s Disease and Movement Disorders, Department of Neurology, University of Cincinnati College of Medicine, Cincinnati, OH USA; 12 Department of Neurology, Beth Israel Deaconess Medical Center, Harvard Medical School, Boston, MA USA; 13 Department of Medicine, Division of Neurology, Parkinson and Movement Disorders Program and the Complex Neurologic Symptoms Clinic, Kaye Edmonton Clinic, University of Alberta, Edmonton, AL Canada; 14 Interdepartmental Neuroscience Program, Northwestern University, Evanston, IL USA; 15 Kellogg School of Management, Northwestern University, Evanston, IL USA

**Keywords:** Parkinson's disease, History, Human behaviour, Parkinson's disease, History

## Abstract

Recent discoveries support the principle that palliative care may improve the quality of life of patients with Parkinson’s disease and those who care for them. Advance care planning, a component of palliative care, provides a vehicle through which patients, families, and clinicians can collaborate to identify values, goals, and preferences early, as well as throughout the disease trajectory, to facilitate care concordant with patient wishes. While research on this topic is abundant in other life-limiting disorders, particularly in oncology, there is a paucity of data in Parkinson’s disease and related neurological disorders. We review and critically evaluate current practices on advance care planning through the analyses of three bioethical challenges pertinent to Parkinson’s disease and propose recommendations for each.

## Introduction

The improvement of the quality of life of patients and their families is a cornerstone of palliative care. Fundamental to the discipline is the alleviation of pain and the suffering arising from other complex symptoms. To facilitate meeting these objectives, advance care planning (ACP) was created in order to “support adults at any age or stage of health in understanding and sharing their personal values, life goals, and preferences regarding future medical care.”^1^ ACP prepares a patient for making future medical choices, and a surrogate to decide on the patient’s behalf should that capacity be lost.^1^ ACP includes the completion of an advance directive (AD), such as the assignment of a health care power of attorney (HCPOA), or the completion of a living will, or both.^2^ The goal of ACP is to ensure that medical care is in alignment with patients’ values, goals, and preferences.^1^ Prior research demonstrates that planning for the future is associated with improvements in patient satisfaction, lower hospital admission rates, and decreases in psychological comorbidities for the family.^3^

Ethical considerations are critical in ACP, given the interdependent issues of personal identity, capacity, autonomy, consciousness, therapeutic nihilism, and stigma. Therefore, the principles of modern biomedical ethics codified by Beauchamp and Childress^4^ in 1979 serve to guide the process: autonomy, beneficence, non-maleficence, and justice. *Autonomy* highlights the respect of patients’ rights to informed self-determination; *beneficence*, the maximization of goodness whereas *non-maleficence* the protection from harm; and *justice* the equity and fairness on a population level. In conjunction with these principles, modern bioethics is also informed by prior instructive cases and by actions and policies aimed at fostering medicine as a moral enterprise (virtue ethics).^5,6^

Among the quality metrics defined for the care of patients with Parkinson’s disease (PD),^7^ the American Academy of Neurology includes initiation of ACP. Data on implementation are limited. In Kentucky, the rate of completion of living wills is 64%, unrelated to disease severity, and comparable to patients without PD.^8^ In Colorado and Florida, AD completion is 56% and 82%, respectively.^9,10^ In South Korea, despite a discussion prevalence of 71%, AD completion is only 27%.^11^ As a result, HCPOAs have insufficient knowledge about their loved ones’ views on life-sustaining measures.^12^

We analyze the three ethical challenges commonly encountered in clinical practice (Table 1) and propose a path forward for each (Table 2). These include (1) the challenge of ACP introduction before the appearance of cognitive impairment without undermining the therapeutic alliance; (2) the challenge of the patient to choose future medical care that may not reflect their future selves; and (3) the challenge to prepare patients and loved ones for the decision to pursue deep brain stimulation (DBS) with preoperative ACP, given that quality of life, identity, cognitive or psychosocial functions may be markedly different after the procedure.

**Table 1:** Review of ACP studies within people with PD (PwP).

Ref	Question	Methods	Results
^8^	What are the uses of certain ADs in various Parkinsonian patients?	• Cross-sectional survey, administered to PwP, atypical PwP, caregivers, and controls	• PwP were at 2-time relative risk of having a HCPOA when compared to controls • PwP are no more likely than controls to utilize a form of medical orders • 80% of atypical PwP had a living will
^12^	What are the practices of ACP and HCPOA within PD?	• Cross-sectional • 64 spouses or adult children who had been designated as the HCPOA	• Only 38% shared living will with physician • 95% of patients with PD completed living will • 42% of HCPOAs not knowledgeable about patients’ views on one of three life-sustaining measures
^14^	What are PwP perspectives on ACP?	• Phenomenological analyses of interviews with PwP	• PwP feel that ACP is neglected, as the visit’s focus is on medication management, or that the clinician would be unable to impart any assistance
^15^	When do PwP want communication on ACP?	• Cross-sectional survey • 585 surveys administered; 267 surveys returned	• 75% of participants felt they themselves should broach the topic of ACP • 94% of participants wanted information on prognosis and treatment early in the course • Around half wanted communication on documentation on ACP • Patients that completed a form of ACP showed fewer depressive features as diagnosed on the PHQ-2
^17^	What is the decisional capacity for PwP without dementia for goals of care discussions?	• 50 participants with average PD duration of 9.8 years, cross-sectional, survey-based • Recruited from movement clinic • Several constructs administered, including the Montreal Cognitive Assessment, Mini Mental Status Examination, and MacArthur Competence Assessment Test	• A substantial subset of participants had impairments in executive function • Participants expressed a choice, yet a subset exhibited impairments in understanding, appreciating, and reasoning
^21^	What are the perspectives of patient and caregiver on ACP in PD?	• 30 patients, and 30 caregivers, multi-site • Randomized clinical trial of palliative intervention for PD compared to standard of care • Qualitative analysis that generated themes	• Patients and caregivers have varying definitions, some of which are broad, and others which are prescriptive • A variety of personal- and systems-level barriers were identified to engaging in ACP; patients report psychiatric dysfunction as impediments to engaging in ACP; hope of the cure is also listed as a barrier as is the perception that wishes might be not be followed through in the future • Caregivers who were elected as surrogates perceived the need to become actively engaged in the decision-making process or understanding preferences of the individual • Participants felt that the palliative care approach that integrated ACP on a periodic basis was useful; those in the standard treatment arm often felt ACP was a one-time event
^23^	What are PwP preferences for end-of-life care assessed retrospectively using death certificates and POLST forms?	• Death certificates in Oregon analyzed from 2010-2011 • Retrospective	• 1.8% of decedents had PD • 35% had completed a physician orders for life-sustaining treatment (POLST) • No significant differences in preferences voiced on POLST in those with PD and those without PD • PwP, on the whole, are more likely to die at a long-term care facility • Decedents with PD who had a POLST and selected comfort measures only were more likely to die at home
^24^	If autobiographical memory in previous work has shown a linkage to selfhood, what are the qualities of autobiographical memories and episodic-future thinking within PwP?	• 15 PwP on stable anti-Parkinsonian medications • 15 healthy age-matched controls • Autobiographical memory = “I AM” statements, includes re-experiencing certain memories within a spatial and visual setting • Episodic-future thinking = “I WILL” statements, involves pre-experiencing certain ideas within spatial and visual settings • Self-concept fluency = how many concepts generated in 60 s • Strength of self-concept = how many concepts were generated but under no time constraint • Verbal fluency assessed in PwP was within normal limits; no different than controls	• No association between “I AM” and “I WILL” performances and disease duration • For the “I AM” task, self-concept fluency was lower in PwP than in healthy controls • Complexity of the items generated was diminished in PwP, both in the “I AM” and “I WILL” tasks
^27^	Are PwP and hospitalized more likely to have a DNR order?	• 12.7 million records reviewed from national database in 2012 for individuals who were 65 or older and hospitalized in 2012 • Retrospective, utilized ICD codes	• PwP more likely to have a DNR order if hospitalized • Disease burden associated with increased odds ratio of DNR status
^28^	What end-of-life wishes would PwP hypothetically decide upon?	• 136 patients with PD; 60 controls • Utilized Willingness to Accept Life-Sustaining Treatments • No significant differences in demographics • Nearly 80% of PwP with H&Y II or lower • Undertaken in Singapore	• Several variables associated with end-of-life preferences: race, marriage, ethnicity, degree of motor impairments, and knowledge of disease • Identification of religion not associated with pursuit of high-burden, poor prognostic care • Knowledge of PD demonstrated an association with end-of-life decisions. Better knowledge of disease was associated with less willingness to endure high-burden, poor prognostic care • Chinese patients were less willing to select aggressive interventions with poor outcomes • Participants with worse motor function showed a relationship with a willingness to pursue more aggressive end-of-life measures than controls
^35^	Explored longitudinally, what are couples’ satisfactions prior to and following DBS over 18 months?	• 30 PwP (16 male), and 16 spouses • Average age 61 years old • Average time of relationship was 31 years • Had average 1.8 children • Underwent bilateral subthalamic stimulation • Utilized MSS-14, HAD-D, HAD-A • Repeated measurements: 2 weeks before DBS, then after DBS at 6 months, 12 months, and 18 months	• How patients rated their satisfaction with their spouse was not associated with scores on depression or anxiety constructs • Greatest decline in couple satisfactions cores occurs during the first year following DBS; thereafter, rate of couple satisfaction—from 12 months to 18 months—is much less so than in the first 12 months
^37^	What effect does subthalamic stimulation have on social adjustment?	• 29 PwP • Before DBS and 1.5 and 2 years after • PDQ-39, social adjustment scale, disability, cognition and psychiatric constructs • Unstructured interviews	• Half of couples were already in marital crisis before surgery • Around 42% of couples who had no evidence of marital crisis before surgery went onto develop a crisis following the operation • 3 PwP divorced during the study • One-third of spouses developed depression after surgery
^38^	Why is it that doctors are happy with the improvements after DBS but the patients apparently “less so”?	• 29 PwP • Semi-structured interviews • Psychosocial assessment undertaken before DBS and 24 months thereafter	• Motor symptoms improved after 24 months (between 60 and 70% reduction in UPDRS-III and UPDRS-IV scores) • Dimensions that were often worse within the social adjustment scale included domains of work and spousal relations
^40^	Does a psychosocial intervention lessen some of the social distress experienced by patients and caregivers during the perioperative period following DBS?	• 19 patients, 7 sessions (4 before DBS, 3 after), each 2 hours, what DBS entailed, counseling on interpersonal and spousal relationships • Randomized 10 to receive the standard of care; the other 9 received the standard of care and augmented with psychosocial interventions • Social Adjustment Scale construct completed 12 months and 24 months following the surgery	• At 12 months after DBS, no significant difference: 29% of patients assigned to receive standard of care plus psychosocial intervention reported further suffering in at least one category within the social adjustment scale • At 24 months after DBS, there was a significant difference between groups (*p* = 0.015): 14% of patients assigned to receive standard of care plus psychosocial intervention reported continuing suffering in at least one category within the social adjustment scale; 80% of patients assigned to receive standard of care reported at least one domain in which they suffered from continued difficulties as assessed from the social adjustment sale

**Table 2:** Summary of ethical challenges and recommendations.

Ethical challenge	Recommendations
Initiating ACP before cognitive dysfunction without undermining the therapeutic alliance	Normalize that ACP is part of a standard, routine and periodic practice
	Communicate the rationale behind early ACP (e.g., to maximize the chances that the patient is participatory given the risk of future cognitive dysfunction, to reduce surrogate distress, and to ensure that care is goal-concordant)
	Establish specific appointments for ACP
	Emphasize that the practice is dynamic (i.e., not a one-time event)
	Integrate chaplains into discussions
	Explore and address psychosocial distress
	Assure the patient that his or her interests are and will remain the priority
Conceptualizing past, current, and future selves to ensure that care is goal-concordant	Explore the patient’s values early in the course of the doctor-patient relationship
	Investigate pertinent elements to the patient’s personhood (e.g., hobbies, professions, meaning, relationships, perspectives on medicine/illness)
	Ensure that ACP discussions are accompanied by loved ones who understand the patient’s history, values and preferences
Preparing patients and caregivers for future decisions across a variety of outcomes for DBS	Inform the patient about all probable outcomes
	Elicit preferences for life and care in the current clinical situation with and without DBS
	Explore and support the needs of the caregivers
	Develop a preoperative decision aid to streamline the process

## Initiating ACP before cognitive dysfunction without undermining the therapeutic alliance

PD is a progressive disease associated with dementia in up to 80% of patients in advanced stages.^13^ This generates the challenge of introducing ACP at a time when cognitive function is preserved (*beneficence*) without jeopardizing the therapeutic alliance (*non-maleficence*). Some neurologists feel obligated to discuss ACP in patients with PD—and these patients want their clinicians to do so.^10,14,15^ Fewer than 10% of neurologists initiate such discussions around the time of diagnosis.^16^ Almost half of the clinicians delay the ACP conversations until cognitive decline manifests.^16^ At that point, patients with PD are unlikely to have the capacity to participate fully in these decisions.^17^

Neurologists may perceive that raising the topic of ACP will amplify suffering, threaten hope, or cause a perception of abandonment and, therefore, might delay such discussion until an episode of acute clinical decline.^16,18^ For patients, barriers to engaging in ACP include concerns that clinicians may subsequently provide lesser quality of care or fears that such discussions are akin to giving up.^19–21^ Because PD is a disease where many will experience disease-related complications resulting in hospitalization, institutionalization, and death, the window in which patients have the capacity to make future medical decisions may be relatively narrow.^17^

Even in the absence of dementia, ACP discussions may be affected. Indeed, a study^17^ showed that a substantial subset of patients with PD, with a mean disease duration of 9.8 years and a Montreal Cognitive Assessment score of 24.5 (close to normal range), exhibited impaired decisional capacity in domains of understanding, appreciating, and reasoning. These findings create a compelling case to begin ACP as early as possible, around the time of diagnosis.^10^ The misguided belief that avoidance of ACP discussions will spare patients from unnecessary emotional distress is at odds with the lower depressive symptomatology ascertained at AD completion.^15^

Addressing barriers to ACP includes establishing specific appointments for ACP, integrating chaplains into the discussions, and treating psychological distress (Lum et al. also offers specific, concrete steps).^2^ Preserving the therapeutic alliance requires communicating the rationale behind early ACP as part of routine and periodic practice, articulating that patients’ interests are the priority, and explaining that ACP neither reduces the intensity or quality of care, nor suggests impending decline.^20^ Transparent reasoning behind early ACP is, therefore, essential. Deferring medical decisions to surrogates when patients are unable to decide for themselves may lead to surrogate distress or interventions that are not concordant with patient wishes.^22^ For example, PD decedents who specified a preference for comfort-focused care were more likely to die at home than those who had not articulated such wishes.^23^

The above can be achieved effectively if ACP becomes a routine part of a practice, integrated into the standard of care, and clarified to represent a flexible rather than a one-time endeavor. This approach may help normalize the conversation for both patient and physician. Communication that the process is dynamic might allay concerns of post decision dissonance and allow patients to focus more on their current goals and values. In practice, the ACP discussion is divided into what the patient wants *now* and what they might want *in the future* if certain health outcomes are reached (e.g., severe dementia or loss of capacity). A frequent review of goals throughout the disease is expected. Since a patient’s decision-making capacity may be greatly affected as the disease progresses, the clinician can review the information obtained overtime to help surrogates infer care preferences in evolving circumstances.

## Conceptualization of past, current, and future selves

Whereas the former challenge relates to the clinician’s timing of ACP discussions to *make* decisions, another challenge is to enable the patient to make *the best* decisions. ACP requires reflection on past, present, and foreseeable selves.^24,25^ Impairments in cognitive flexibility, seen early within the disease course, result in patients who may struggle to envision their future selves.^26^ In contrast to prior work that suggests that patients with chronic disease and poor performance status desire less aggressive measures^27^, patients with PD may wish to pursue high-burden care in the setting of poor prognoses when compared to controls.^28^ *The challenge arises that if patients with PD experience deficits in envisaging, then how can patients and clinicians optimally collaborate so that future care reflects the person holistically?*

A set of philosophical assumptions underpinning this discussion are that the self at time point 1 (t1) should justifiably be able to decide for the self at time point 2 (t2). This relies on an account of personhood, which dictates that the self at t1 is the same self at t2. Suppose the preferences of the self at t2 change, in the context of PD. Does this self at t2 not really know what the self at t2 wants? What permits the self at t1 to override the self at t2?

A recent study highlights this challenge by showing that patients with PD (1) may experience deficits in envisaging early in the disease, and (2) that this deficit is not related to disease severity.^24^ This was tested by assessing autobiographical memory (AM), and episodic-future thinking (EFT) as these are likely critical to the cognitive processes involved in decision-making and social functioning and can be markedly impaired in a host of neurological diseases.^24^ In the study, researchers explored the abilities of patients with PD to access AM, and EFT.^24^ AM involved re-experiencing memories, and EFT involved the pre-experiencing of foreseeable situations. Compared to healthy controls, participants generated fewer concepts and with less complexity for the AM task; complexity was also diminished during EFT.

Because of these findings, we may be inclined to view the t2 self as a less-robust or diseased version of t1, but perhaps we ought to be viewing the t2 self as an entirely different individual distinct from the t1 self. The “disability paradox” corroborates this notion: individuals who imagine a future disability tend to assume the quality of life would be lower than the patients who have that disability rate their quality of life.^29,30^ This discrepancy provides empirical data that the t1 and t2 selves often have different values, independent of incapacity or cognitive dysfunction. As the self at t2 is not completely independent from the self at t1, it follows that if the t2 self is severely impaired and inconsistent, then allowing an AD or HCPOA to serve as the voice not only of t1 but also of t2 may be sensible. Yet if, on the other hand, the t2 self has some impairment but is consistent and compelling or demonstrates a quality of life greater than what t1 predicted, a reconsideration is in order.

While alterations of personhood complicate ACP, “projection bias” often also confounds the process. Projection bias is a cognitive error in which emotional states alter the ability to consider future events rationally.^31^ For instance, cancer patients’ willingness to live varied by 30% throughout a given day in tandem with psychological states.^31^ Present reflections may thereby supersede more calculated analyses of past or future selves.^28,31^ Clinicians may be unaware of the transient nature of a particular preference or may be unable to operationalize those preferences.^22,31^ This may have vexing implications when deciding on potential future life-sustaining measures in a disease where mood often fluctuates in ON- and OFF-medication states.^32^

Two approaches of virtue ethics may help surmount the challenges: (1) incorporating loved ones in the ACP process so that they may provide additional perspectives on the patient, and (2) getting to know the patient well.

Clinicians should, first, encourage patients with PD at all stages of the disease to undertake ACP accompanied by loved ones who understand their history, values, and preferences. A collaborative process that encourages patients to discuss their wishes with others may forestall the intrusion of cognitive errors during medical decision-making. In addition, the incorporation of others’ views can reveal certain “blind spots” that might have otherwise been overlooked. Second, clinicians ought to become acquainted with patients at the outset. What hobbies or professions did they pursue? What brought joy and meaning? How did they react when others were ill? Did they always participate in their medical care, or did they prefer not to visit doctors? Some of these answers help explore “personhood” and may help shed light on approaches to decision-making. Taken together, these strategies will further enhance the clinician’s ability to make a holistic, personalized recommendation rather than a general recommendation made for all patients.

## Preoperative ACP for DBS

Our third challenge relates to the potential missed opportunity for preoperative ACP before DBS surgery that can inform future medical decision-making, including potential perioperative complications. The eligibility mechanism for selection of DBS surgery involves examination of the motor, cognitive, and psychiatric states. What is often overlooked is that patients and families may receive inadequate preparation on the potential risks, range of outcomes, and side-effects of surgery. *The challenge arises that if the quality of life, identity, cognitive, or psychosocial functions may markedly change after DBS, then how should patients and caregivers adequately prepare themselves for future decisions across a variety of outcomes?*

Currently, ACP is not considered a best practice or standard for preoperative DBS planning.^16,33^ The current standard of care, therefore, elevates the likelihood of enabling discordant care and dysfunctional social situations.^34–38^ Following DBS, neuropsychiatric features such as suicidal ideation, apathy, mania, and delirium may appear, which may be refractory to adjustments in electrical stimulation or pharmacotherapy.^39^ Intracranial hemorrhage or hardware malfunction, while uncommon, may also occur.^39^ The steepest decline in couple satisfaction, for example, occurs during the first year after DBS surgery;^35^ within 2 years, one-third of spouses develop depression, and 42% of couples without prior histories of marital crises develop them.^37^

Only preliminary data is available regarding mitigating strategies.^40^ DBS is ideally suited for a well-designed, shared-decision-making tool such as a preoperative decision aid.^41^ A Cochrane review of trials of decision aids in other settings found that such aids help reduce decision conflict and ameliorate future regret while assuring that patients are well informed about all outcomes and that the treatments are consistent with their values.^42^ A balanced decision aid designed for patients with PD around DBS would need to be carefully designed so that it is both accurate and accessible for understanding. It would also need to elicit the individual’s preferences for life and care in the current situation with and without DBS as well as acknowledge the needs of the caregivers.

## Implications for future research and innovation

The development of shared-decision-making aids will be vital to improving ACP, particularly tools that include stories from patients and families describing how satisfied they are with their decisions, and why they feel that way. It has not escaped our notice that while no substitute exists for frequent conversations with the individual patients and families, advances in decision-making and data analytics create an opportunity to apply artificial intelligence to ACP for neurological diseases. Efforts examining the accuracy of surrogates in predicting patients’ desires show that, on average, they can correctly predict choices at a rate between 59.3% and 68%.^22,43^ In contrast, tools that incorporate computer guidance may assist both patients and clinicians to improve conversations^44^ and predict choices.^45–48^

Methodologically, the integration of behavioral economics and data science may improve predictive capacity for future choices.^45^ Longitudinal data collection of the goals, behaviors, beliefs, and affective states is likely to later aid in predicting patient-centered communication styles, preferences, and preferred clinical choices. Foreseeably, an auto-generated report, personalized for a given patient, that uses natural language technologies would be dynamic and reflective of inputs captured over time. Such a user-friendly document would allow for dissemination to patients, their loved ones, and the clinical team to assist in their deliberations during times of clinical change. These tools can allow loved ones and clinicians to develop a more comprehensive understanding of the patient and predict their preferences in future stages. Additionally, if a surrogate is required to decide on behalf of a patient, then a machine-aided interpretation of “what the patient would want”—with certain predicted likelihoods attached to each option—may assist with such a decision.

Further research is required to measure perceptions of the accuracy of decisions near the end-of-life and to determine whether these decisions are concordant with intended care preferences. Such inquiry ought to be aligned with the principles of modern biomedical ethics, while based on cases that are tailored to neural disorders and their circumstances. This should be ideally coupled with efforts to better understand the preferences, practices, and attitudes of movement disorder neurologists surrounding the integration of palliative care and ACP into clinical practice. Developing and validating improved shared-decision-making strategies, aids, and vignettes of clinical outcomes will be crucial to ensure that patient preferences and values are optimally captured and honored.
